# Childhood‐onset systemic lupus erythematosus with trisomy X and the increased risk for bone complications: a case report

**DOI:** 10.1186/s12969-021-00507-3

**Published:** 2021-02-23

**Authors:** Susumu Yamazaki, Yuko Akutsu, Asami Shimbo, Masaki Shimizu, Yuko Segawa, Masaaki Mori

**Affiliations:** 1 Department of Lifetime Clinical Immunology, Graduate School of Medical and Dental Sciences, Tokyo Medical and Dental University, 1-5-45, Yushima, Bunkyo-ku, 113-8519 Tokyo, Japan; 2 Department of Pediatrics and Adolescent Medicine, Juntendo University Graduate School of Medicine, Tokyo, Japan; 3 Department of Pediatrics and Developmental Biology, Perinatal and Maternal Medicine, Graduate School of Medical and Dental Sciences, Tokyo Medical and Dental University, Tokyo, Japan; 4 Department of Orthopedic Surgery, Tokyo Medical and Dental University, Tokyo, Japan

**Keywords:** Trisomy X, Systematic lupus erythematosus, Corticosteroids, Avascular necrosis, Osteoporosis

## Abstract

**Background:**

Systemic lupus erythematosus is a multi-organ inflammatory autoimmune disease; immune complexes are part of the pathogenesis, but not entirely responsible. Trisomy X is the most common female chromosomal abnormality and the role of an additional X chromosome in the development of systemic lupus erythematosus is well recognized. However, the potential complications and optimal management of childhood lupus with trisomy X remain unclear. Herein, we describe a case of childhood-onset systemic lupus erythematosus associated with severe bone complications presumably secondary to trisomy X.

**Case presentation:**

A 16-year-old Japanese girl was diagnosed with childhood-onset systemic lupus erythematosus and trisomy X. A chromosomal abnormality (47, XXX) was incidentally identified on bone marrow examination initially done to determine the cause of pancytopenia. She had a persistent headache, fever　for six days, diffuse hair loss, mucosal ulcers, butterfly eruptions, and palmar erythema. Furthermore, thrombocytopenia, anemia, and erythrocyte fragmentation were detected, suggesting secondary thrombotic microangiopathy. She was initially treated with intravenous methylprednisolone pulse therapy and prescribed monthly cyclophosphamide for severe disease activity, prednisolone, mycophenolate mofetil, and hydroxychloroquine as remission maintenance drugs. She developed generalized extremity pain that had been worsening throughout the disease. Extremity magnetic resonance imaging performed 12 months after the treatment onset revealed multifocal avascular necrosis, and dual-energy X-ray absorptiometry revealed further decreased bone mineral density. High plasma levels of factor VIII were detected by additional tests for coagulation functions, and we suspected the possibility that factor VIII might cause avascular necrosis due to thrombosis. Currently, she is being treated with prednisolone and MMF for SLE. However, her extremity pain has not been managed effectively even under the administration of non-steroidal anti-inflammatory drugs and pregabalin.

**Conclusions:**

An additional X chromosome has been reported to be associated with factor VIII and osteoporosis. Additionally, elevated plasma levels of FVIII is the risk factors for thrombosis, which leads to the risk of avascular necrosis. Patients with systemic lupus erythematosus complicated by trisomy X might be at a higher risk of avascular necrosis and osteoporosis that can also manifest in childhood systemic lupus erythematosus.

## Background

Systemic lupus erythematosus (SLE) is a multi-organ inflammatory autoimmune disease of unknown cause; immune complexes are part of the pathogenesis but not entirely responsible. If SLE develops before 18 years of age, it is classified as childhood-onset systemic lupus erythematosus (cSLE) [1]. Avascular necrosis (AVN) is a well-recognized complication of systemic lupus erythematosus (SLE), but the risk of AVN is usually lower in children than in adults. The prevalence of AVN in patients with SLE ranges between 10 % and 15 % [2]. Conversely, AVN prevalence in cSLE ranges between 5.4 % and 8.4 % [3–5].

The importance of the X chromosome in the pathogenesis of systemic lupus erythematosus (SLE) is well recognized, but its role in the development of bone complications remains unclear. Trisomy X is the most common female chromosomal abnormality, occurring in approximately 1 in 1,000 female births; most individuals are only mildly affected or asymptomatic [6]. The risk of SLE in Klinefelter’s syndrome is similar to that of normal females [7], and the prevalence of SLE in trisomy X is 2.5 times higher than in chromosomally normal females [8]. However, the studies of the clinical manifestations of SLE in trisomy/polysomy X have been scarce, and the bone complications have not been mentioned in any of them [9–11].

Herein, we report a case of cSLE in a female patient with trisomy X that developed severe bone complications.

## Case presentation

The case described is of a 16-year-old Japanese girl who had no relevant family history. She had a medical history of attention deficit hyperactivity disorder (ADHD) and had been on atomoxetine since she was 12 years. She had developed stomatitis at the age of 12, alopecia at the age of 14, and butterfly erythema vulgaris at the age of 15. At the age of 16, she was referred to our hospital for suspected cSLE.

**Fig. 1:**
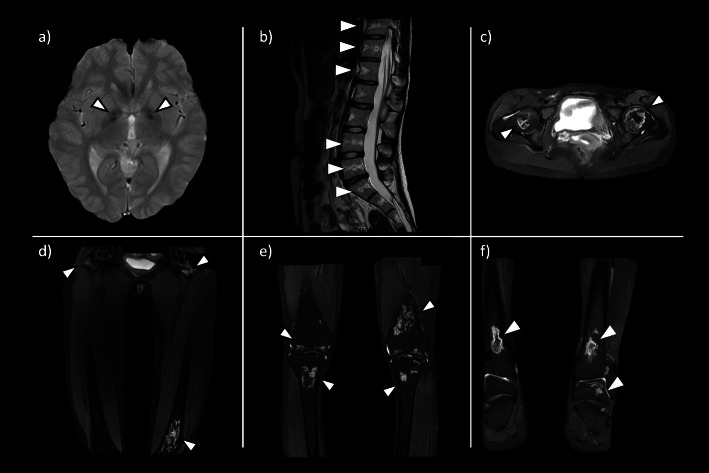
MRI (T2 weighted Image) findings were inconsistent with the patient’s age and revealed multiple sites of AVNSuspected calcification of the globus pallidus (**a**) and fatty changes in the lumbar spine (**b**: sagittal view), which are usually found in the elderly. AVN is seen in femoral head (**c**: axial view, **d**: coronal view); distal femur and proximal tibia (**e**: coronal view); distal tibia and talus (f: coronal view) AVN, avascular necrosis; MRI, magnetic resonance imaging

On physical examination at first presentation, the patient’s height and weight were 169.1 cm (+ 2.2 standard deviations above age average) and 44.2 kg, respectively; the tall stature was suggestive of trisomy X, but the associated facial features, such as epicanthal folds or hypertelorism, were not observed. She had a persistent headache, fever for six days, diffuse hair loss, mucosal ulcers, butterfly eruptions, and palmar erythema. Blood tests revealed pancytopenia (total white blood cell count: 3,400/µL; lymphocyte count: 958/µL;　hemoglobin level: 7.9 g/dL; platelet count: 149,000/µL), In addition to thrombocytopenia and anemia, erythrocyte fragmentation (7.0 ‰; normal value: <1.2 ‰ [12]), elevated lactate dehydrogenase (355 IU/L), serum creatinine levels (0.91 mg/dL), low haptoglobin (< 10 mg/dL), and negative Coombs tests were observed, which were suggestive of secondary thrombotic microangiopathy(TMA); ADAMTS13 activity was normal.　Concerning other SLE findings, low complement levels (C3: 25 mg/dL; C4: 2 mg/dL; CH50: 10 U/mL), and normal C-reactive protein levels (0.3 mg/dL) were revealed. The patient tested positive for the following autoantibodies: anti-nuclear antibody titer > 1:1280, homogeneous and speckled pattern; anti-DNA antibody 520 IU/mL; anti-double-stranded DNA antibody 1,010 IU/mL; anti-Smith antibody > 1:32; anti-U1 ribonucleoprotein antibody > 1:256. Tests for anti-SS-A antibody, anti-Scl-70 antibody, PR3-antineutrophil cytoplasmic antibody (ANCA), myeloperoxidase-ANCA, anti-cardiolipin antibody (IgG), lupus anticoagulant, and anticardiolipin/beta2-glycoprotein I complex antibodies were negative. Urine analysis showed proteinuria (2.4 g/day), mixed cellular casts, and red blood cells; kidney biopsy was not performed, since urinary analysis results improved soon after treatment. Underlying infectious disease was ruled out by blood culture and whole-body computed tomography scan. To exclude malignancy due to pancytopenia, a bone marrow examination was performed. The result showed normocellular marrow and denied leukemia and myelodysplastic syndrome, but a chromosomal abnormality (47, XXX) was incidentally identified. Consequently, the patient was diagnosed with cSLE with trisomy X based on the American College of Rheumatology revised criteria [13], and was classified as having high disease activity, as the SLE disease activity index (SLEDAI) score was 27 [14]. There were two unusual findings for SLE. One was palladium calcification detected by the cranial magnetic resonance imaging (MRI) (Fig. 1a), and another was low bone mineral density detected by the dual-energy X-ray absorptiometry (DEXA) (lumbar spine: 0.972 g/cm2; Z-score − 1.7), which are commonly observed in the elderly.

**Fig. 2:**
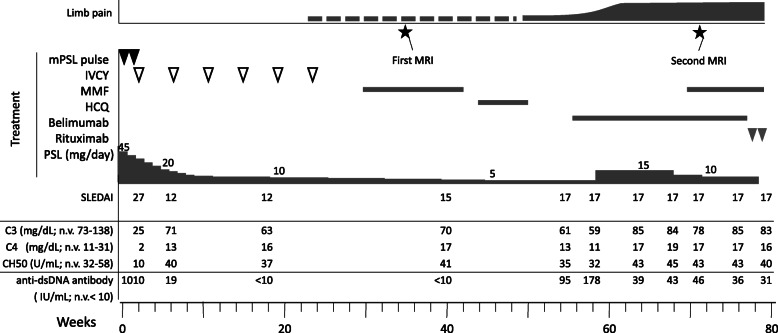
Clinical course of the patient Black triangles show mPSL pulse (one course; mPSL 1 g/day for 3 days and maintenance therapy with prednisolone 1 mg/kg/day for 4 days). White triangles show monthly cyclophosphamide treatment 0.5 g/m^2^ (total six times). The first course of mycophenolate mofetil and hydroxychloroquine were discontinued owing to leukopenia and alopecia, respectively. From an early stage in the disease course, extremity pain had developed and worsened HCQ, hydroxychloroquine; IVCY, intravenous cyclophosphamide; MMF, mycophenolate mofetil; mPSL, methylprednisolone; PSL, prednisolone; SLEDAI, systemic lupus erythematosus disease activity index

Due to high disease activity and suspected TMA complications, the patient chose a strong treatment method. The course of treatment is shown in Fig. 2. She was initially treated with two courses of intravenous methylprednisolone pulse therapy (1 g/day for 3 days and maintenance therapy with prednisolone 1 mg/kg/day for 4 days) with heparinization (150 IU/kg/day). Thereafter, monthly cyclophosphamide treatment was added (0.5 g/m^2),^ and prednisolone was tapered. Alfacalcidol (0.5 µg/day) was administered from onset to prevent osteoporosis due to steroids’ side effects. Mycophenolate mofetil (MMF) (1500 mg/day) and hydroxychloroquine (200 mg/day) were added for the maintenance of remission, but were discontinued secondary to their adverse effects of leukopenia and alopecia, respectively; these symptoms abated after the withdrawal of these medications. Subsequently, belimumab was added as a remission maintenance drug, but no obvious effect was observed, and prednisolone dosage was increased up to 15 mg/day to control headache and elevated anti-dsDNA antibody. Generalized extremity pain developed early and worsened throughout the disease. Her visual analog scale of extremity pain score was always 9–10/10 even under administration of non-steroidal anti-inflammatory drugs, but differed from the doctor’s impression; because she could walk and bend knees naturally. Extremity MRI performed 6 months after the treatment onset was normal. However, a second MRI performed 6 months later revealed multifocal avascular necrosis (AVN) and the increased volume of adipose tissue in the bone marrow of the spine, similar to what is observed in the elderly [15] (Fig. 1b-f). Since the location of pain spread beyond the AVN position and unnaturally high visual analog scale score was persistent, it was determined that the causes of pain were attributable to a mixture of AVN and complex regional pain syndrome. Hence, pregabalin was added, but no effect was observed, and the pain persisted.

Additional tests for coagulation defects were performed because one of the proposed mechanisms for vascular interruption in AVN is coagulation/ thrombus formation [4, 5]. Prothrombin time, activated partial prothrombin time, D-dimer levels, protein C and protein S activation, and antithrombin III activity were normal. However, the plasma levels of factor VIII (FVIII) and VWF antigen (VWF: Ag) were elevated (FVIII: 192.4 %, normal range 78–165 %; VWF: Ag > 201 %, normal range 50–150 %). These findings ruled out congenital thrombotic disorders such as protein C/S deficiency but revealed that the potential thrombotic condition might be caused by high levels of FVIII [16] and VWF: Ag [17]. Additionally, further deterioration and a mildly decreased bone mineral density were observed on the second DEXA (lumbar spine: 0.956 g/cm^2^; Z-score − 1.8). MMF was restarted for the concerns of ongoing deterioration, and rituximab was added to reduce steroid-related adverse effects, such as bone complications.

Currently, she is being treated with prednisolone and MMF for SLE. However, her extremity pain has not been managed effectively.

## Discussion and conclusions

We have described a case of cSLE in a patient with trisomy X complicated by AVN and a decreased bone mineral density. In our patient, the development of these complications may have been related to an additional X chromosome.

It is possible that thrombosis due to interactions between FVIII encoded by the X chromosome and VFW might have caused AVN in our patient. Elevated plasma levels of FVIII [16] and VWF: Ag [17] are the risk factors for arterial and venous thrombosis, and a recent study suggested that their levels correlate [18]. Since the gene encoding FVIII is located on the long arm of the X chromosome [19], trisomy X patients might have possibly induced thrombosis due to the overexpression of FVIII. To support this hypothesis, the tendency for females to have higher levels of FVIII than males [20] might suggest an effect of the X chromosome on FVIII. In addition, a case of a severe leg ulcer in XXXXY syndrome due to elevated FVIII was previously reported [21]. A case-based review of SLE in female polysomy X reported that four out of five cases developed arthritis [9], which might be attributed to AVN.　On the other hand, there was a report that FVIII levels increased even in Turner syndrome patients with portal vein thrombosis [22]. However, in that report, FVIII and VWF were not different between 25 Turner syndrome patients without thrombosis and 25 normal girls. Hence the high-level FVIII in Turner syndrome patients might be regarded as a consequence of just thrombosis, and not the dose of X chromosome.

Moreover, an additional X chromosome can elevate the risk for osteoporosis. Given that Klinefelter syndrome has been associated with an increased risk of osteoporosis [7], trisomy X may similarly follow suit. In addition, some trisomy X patients can develop premature ovarian failure, which is also a risk factor for osteoporosis [6]. Although our patient had not developed premature ovarian failure yet (progesterone and estradiol levels were normal), she already had a low bone mineral density before the start of the treatment, which might have reflected the characteristics of trisomy X. Since the use of corticosteroids is a well-known predisposing factor for osteoporosis [23], extra care should be taken when corticosteroid therapy is prescribed for the trisomy X patients compared to chromosomally normal females.

The hypothesis that an additional X chromosome might induce AVN due to thrombosis by overexpression of *FVIII* is derived by findings from only this single case, and this is our limitation. Of the 72 SLE patients, 32 had AVN, and 14 were reported to have multiple AVN in 4 or more locations [24]. In our report, although multiple AVN can be explained as just a potential complication of SLE, it would be rarely explained as cSLE in our experience. There were no reports of FVIII statistics in trisomy X; only one case report has reported this, as mentioned above [21]. Although not all trisomy X cases develop into thrombosis, and most are asymptomatic, in the case of SLE with trisomy X, we might need to be concerned about FVIII and bone complications.

In summary, it is important to consider the risk of AVN and osteoporosis in SLE patients with trisomy X more so than in chromosomally normal females, even in the case of a childhood onset.

All authors read and approved the final manuscript.

## Data Availability

Not applicable.

## Abbreviations

SLE: Systemic lupus erythematosus
cSLE: Childhood-onset systemic lupus erythematosus
PSL: Prednisolone
AVN: Avascular necrosis
ANCA: Antineutrophil cytoplasmic antibody
MRI: Magnetic resonance imaging
MMF: Mycophenolate mofetil
FVIII: Factor VIII
VWF: Ag Von Willebrand factor antigen

## References

[1] Silva CA, Avcin T, Brunner HI (2012). Taxonomy for systemic lupus erythematosus with onset before adulthood. Arthritis Care Res.

[2] Hussein S, Suitner M, Béland-Bonenfant S, Baril-Dionne A, Vandermeer B, Santesso N (2018). Monitoring of Osteonecrosis in Systemic Lupus Erythematosus: A Systematic Review and Metaanalysis. J Rheum.

[3] Ravelli A, Duarte-Salazar C, Buratti S, Reiff A, Bernstein B, Maldonado-Velazquez MR (2003). Assessment of damage in juvenile-onset systemic lupus erythematosus: a multicenter cohort study. Arthritis Rheum.

[4] Yang Y, Kumar S, Lim LS, Silverman ED, Levy DM (2015). Risk factors for symptomatic avascular necrosis in childhood-onset systemic lupus erythematosus. J Rheum.

[5] Gurion R, Tangpricha V, Yow E, Schanberg LE, McComsey GA, Robinson AB (2015). Avascular necrosis in pediatric systemic lupus erythematosus: a brief report and review of the literature. Pediatr Rheumatol Online J.

[6] Tartaglia NR, Howell S, Sutherland A, Wilson R, Wilson L (2010). A review of trisomy X (47,XXX). Orphanet J Rare Dis.

[7] Scofield RH, Bruner GR, Namjou B, Kimberly RP, Ramsey-Goldman R, Petri M (2008). Klinefelter’s syndrome (47,XXY) in male systemic lupus erythematosus patients: support for the notion of a gene-dose effect from the X chromosome. Arthritis Rheum.

[8] Liu K, Kurien BT, Zimmerman SL, Kaufman KM, Taft DH, Kottyan LC (2016). X Chromosome dose and sex bias in autoimmune diseases: increased prevalence of 47,XXX in systemic lupus erythematosus and Sjögren’s syndrome. Arthritis Rheumatol.

[9] Iwamoto T, Fujimoto M, Ikeda K, Saku A, Makita S, Furuta S (2019). Manifestations of systemic lupus erythematosus in female patients with polysomy X: possible roles of chromosome X. Mod Rheumatol.

[10] Slae M, Heshin-Bekenstein M, Simckes A, Heimer G, Engelhard D, Eisenstein EM (2014). Female polysomy-X and systemic lupus erythematosus. Semin Arthritis Rheum.

[11] Barbosa FB, Sinicato NA, Julio PR, Londe AC, Marini R, Gil-da-Silva-Lopes VL (2020). Trisomy X in a patient with childhood-onset systemic lupus erythematosus. J Transl Autoimmun.

[12] Zeigler ZR, Rosenfeld CS, Andrews DF, Nemunaitis J, Raymond JM, Shadduck RK, et al. Plasma von Willebrand Factor Antigen (vWF:AG) and thrombomodulin (TM) levels in Adult Thrombotic Thrombocytopenic Purpura/Hemolytic Uremic Syndromes (TTP/HUS) and bone marrow transplant-associated thrombotic microangiopathy (BMT-TM). Am J Hematol. 1996;53:213–20. 10.1002/(SICI)1096-8652(199612)53:4<213::AID-AJH1>3.0.CO;2-0 8948657

[13] Hochberg MC (1997). Updating the American College of Rheumatology revised criteria for the classification of systemic lupus erythematosus. Arthritis rheumatism.

[14] Bombardier C, Gladman DD, Urowitz MB, Caron D, Chang CH (1992). Derivation of the SLEDAI. A disease activity index for lupus patients. The Committee on Prognosis Studies in SLE. Arthritis rheumatism.

[15] Justesen J, Stenderup K, Ebbesen EN, Mosekilde L, Steiniche T, Kassem M (2001). Adipocyte tissue volume in bone marrow is increased with aging and in patients with osteoporosis. Biogerontology.

[16] Kyrle PA, Minar E, Hirschl M, Bialonczyk C, Stain M, Schneider B (2000). High plasma levels of factor VIII and the risk of recurrent venous thromboembolism. N Engl J Med.

[17] Swystun LL, Lillicrap D (2018). Genetic regulation of plasma von Willebrand factor levels in health and disease. J Thromb Haemost.

[18] Song J, Chen F, Campos M, Bolgiano D, Houck K, Chambless LE (2015). Quantitative influence of ABO blood groups on factor VIII and its ratio to von Willebrand factor, novel observations from an ARIC study of 11,673 subjects. PLoS One.

[19] Thompson AR (2003). Structure and function of the factor VIII gene and protein. Semin Thromb Hemost.

[20] Conlan MG, Folsom AR, Finch A, Davis CE, Sorlie P, Marcucci G (1993). Associations of factor VIII and von Willebrand factor with age, race, sex, and risk factors for atherosclerosis. The Atherosclerosis Risk in Communities (ARIC) Study. Thromb Haemost.

[21] Akiyama M, Ueno T, Niimi Y, Sakai N, Kawana S (2011). Leg ulcer in a patient with 49, XXXXY syndrome. J Dermatol.

[22] Kopacek Zilz C, Keller Brenner J, Elnecave RH (2006). Portal vein thrombosis and high factor VIII in Turner syndrome. Hormone research.

[23] Buckley L, Guyatt G, Fink HA, Cannon M, Grossman J, Hansen KE (2017). 2017 American college of rheumatology guideline for the prevention and treatment of glucocorticoid-induced osteoporosis. Arthritis Care Res (Hoboken).

[24] Oinuma K, Harada Y, Nawata Y, Takabayashi K, Abe I, Kamikawa K, et al. Osteonecrosis in patients with systemic lupus erythematosus develops very early after starting high dose corticosteroid treatment. Ann Rheum Dis. 2001;60(12):1145–8. 10.1136/ard.60.12.1145 PMC1753447 11709458

